# Sex-Biased lncRNA Signature in Fetal Growth Restriction (FGR)

**DOI:** 10.3390/cells10040921

**Published:** 2021-04-16

**Authors:** Aleksandra Lipka, Jan Pawel Jastrzebski, Lukasz Paukszto, Karol Gustaw Makowczenko, Elzbieta Lopienska-Biernat, Marek Gowkielewicz, Ewa Lepiarczyk, Marta Wiszpolska, Mariusz Krzysztof Majewski, Marta Majewska

**Affiliations:** 1 Department of Gynecology and Obstetrics, School of Medicine, Collegium Medicum, University of Warmia and Mazury in Olsztyn, 10-561 Olsztyn, Poland; aleksandra.lipka@uwm.edu.pl (A.L.); marekgowkielewicz@gmail.com (M.G.); 2 Bioinformatics Core Facility, Faculty of Biology and Biotechnology, University of Warmia and Mazury in Olsztyn, 10-719 Olsztyn, Poland; pauk24@gmail.com; 3 Department of Plant Physiology, Genetics and Biotechnology, Faculty of Biology and Biotechnology, University of Warmia and Mazury in Olsztyn, 10-719 Olsztyn, Poland; 4 Department of Animal Anatomy and Physiology, Faculty of Biology and Biotechnology, University of Warmia and Mazury in Olsztyn, 10-719 Olsztyn, Poland; karol.makowczenko@gmail.com; 5 Department of Biochemistry, Faculty of Biology and Biotechnology, University of Warmia and Mazury in Olsztyn, 10-719 Olsztyn-Kortowo, Poland; ela.lopienska@uwm.edu.pl; 6 Department of Human Physiology and Pathophysiology, School of Medicine, Collegium Medicum, University of Warmia and Mazury in Olsztyn, 10-082 Olsztyn, Poland; ewa.lepiarczyk@uwm.edu.pl (E.L.); marta.wiszpolska@uwm.edu.pl (M.W.); mariusz.majewski@uwm.edu.pl (M.K.M.); marta.majewska@uwm.edu.pl (M.M.)

**Keywords:** FGR, placenta, RNA-seq, lncRNA, transcriptome

## Abstract

Impaired fetal growth is one of the most important causes of prematurity, stillbirth and infant mortality. The pathogenesis of idiopathic fetal growth restriction (FGR) is poorly understood but is thought to be multifactorial and comprise a range of genetic causes. This research aimed to investigate non-coding RNAs (lncRNAs) in the placentas of male and female fetuses affected by FGR. RNA-Seq data were analyzed to detect lncRNAs, their potential target genes and circular RNAs (circRNAs); a differential analysis was also performed. The multilevel bioinformatic analysis enabled the detection of 23,137 placental lncRNAs and 4263 of them were classified as novel. In FGR-affected female fetuses’ placentas (ff-FGR), among 19 transcriptionally active regions (TARs), five differentially expressed lncRNAs (DELs) and 12 differentially expressed protein-coding genes (DEGs) were identified. Within 232 differentially expressed TARs identified in male fetuses (mf-FGR), 33 encompassed novel and 176 known lncRNAs, and 52 DEGs were upregulated, while 180 revealed decreased expression. In ff-FGR *ACTA2-AS1*, lncRNA expression was significantly correlated with five DEGs, and in mf-FGR, 25 TARs were associated with DELs correlated with 157 unique DEGs. Backsplicing circRNA processes were detected in the range of *H19* lncRNA, in both ff- and mf-FGR placentas. The performed global lncRNAs characteristics in terms of fetal sex showed dysregulation of DELs, DEGs and circRNAs that may affect fetus growth and pregnancy outcomes. In female placentas, DELs and DEGs were associated mainly with the vasculature, while in male placentas, disturbed expression predominantly affected immune processes.

## 1. Introduction

Adequate fetal growth and proper functioning of the placenta is a valuable predictor of pregnancy outcome [1]. Hence, impaired fetal growth is one of the most important causes of prematurity, stillbirth and infant mortality [2,3]. The etiology of fetal growth restriction (FGR) is multifactorial and comprises a wide range of various either maternal, fetal, placental or genetic causes [4,5]. Maternal risk factors include infections (TORCH, malaria), poor maternal health (malnutrition, diabetes mellitus, hypertension, anemia, cardiac, hepatic and chronic renal diseases), obesity (BMI > 30), drug abuse, smoking, female age above 35 years and multiple gestations [5,6]. Fetal factors encompass chromosomal abnormalities, as at least 50% of fetuses with trisomy 21, 13 or 18, or Turner’s syndrome are associated with a higher rate of fetal growth restriction [7]. Moreover, specific placental disorders like infarction, fetal vessel thrombosis, preeclampsia (PE), decidual or spiral artery arteritis, chronic villitis and placental hemangioma, as well as umbilical cord abnormalities, like velamentous or marginal cord insertion, also affect FGR initiation [4,8]. While those environmental cues have been associated with the development of FGR, idiopathic aberrations in placental function account for nearly 70% of FGR cases in normally formed fetuses [9]. The pathogenesis of idiopathic FGR is poorly understood but might be the effect of placental insufficiency with interference in nutrient supply, redox balance and energy metabolism [10]. Growth-restricted newborns are susceptible to pulmonary hypertension, hypothermia, pulmonary hemorrhage, and hypo- and hyper-glycemia [4,11] and may suffer from cognitive delay, and neurological and psychiatric disorders in childhood [4,11,12,13,14]. Adults are prone to obesity, hypertension and Type 2 diabetes, as well as neurological, cardiovascular, renal, hepatic and respiratory diseases [14,15,16,17].

With high-throughput sequencing and bioinformatics development, it appears that the number of non-coding RNAs (ncRNAs) greatly exceeds the number of protein-coding genes. Transcripts that are not translated into proteins can be divided into several classes: long non-coding RNAs (lncRNAs), circular RNAs (circRNAs), small non-coding RNAs such as microRNAs (miRNAs), small interfering RNAs (siRNAs), piwi-interacting RNAs (piRNAs), small nucleolar RNAs (snoRNAs) and other short RNAs [18,19]. Transcripts to be classified as lncRNA need to meet criteria regarding length (>200 nt) and a structure that must contain at least two exons. The majority of lncRNAs exist as a single isoform and 98% of them are spliced and 80% have 2–4 exons [20,21]. The genomic localization of the lncRNA may be various, from the introns of protein-coding genes to enhancer-associated RNAs resulting from direct or bidirectional transcription of enhancer elements. Non-coding transcripts located between coding and non-coding genes do not overlap the exons of other genes, which are long intergenic (or intervening) ncRNAs (lincRNAs). Natural antisense transcripts are transcribed for the antisense strand of a coding (or non-coding gene) but their transcription start site is located downstream relative to that of the coding gene [22,23]. As a large ncRNAs class, circRNAs, produced due to non-canonical backsplicing, should also be mentioned. CircRNAs are mostly transcribed from protein-coding genes but may include exons that are skipped during canonical types of alternative splicing, as well as introns [24]. CircRNAs may exert a regulatory function by acting as microRNA or protein inhibitors, which multiplies their biological effects. Moreover, the expression of circRNAs is tissue-specific and plays an important role in the physiological development and pathogenesis of various diseases [25].

There is growing evidence that some differences in placenta efficiency and growth depend on the sex of the fetus [26,27]. Male fetuses grow faster and, at birth, have a greater body length and mass than female fetuses with comparable placenta size [28]. At the cellular level, differences between male and female placentas result from the activity of X- and Y-linked genes that also may have the potential to control and regulate autosomal gene expression [29]. Research performed on physiological human placentas concerning sex-bias comparisons revealed five differentially expressed lncRNAs: *HAND2-AS1*, *XIST*, *RP1-97J1.2*, *AC010084.1* and *TTTY15* [30]. Studies of placentas from FGR-complicated pregnancies identified 28 differentially expressed genes (DEGs), and their functional enrichment annotation indicates that most of them are implicated in inflammation and immune disorder processes related to FGR and PE. Genes that display differential alternative splicing (DAS) events (*S100A13*, *GPR126*, *CTRP1*, and *TFPI*) are mainly implicated in angiogenic-related processes [31]. Investigating the mechanisms regulating placental development and potentially underlying the FGR should be extended with a complete analysis of the lncRNA profile. Studies of single lncRNAs revealed that their specific profile may make a contribution to placenta-associated conditions [32]; however, high-throughput analysis characterizing a complete lncRNA profile in FGR-affected placentas has not yet been performed. As far as it is known, this research is the first step towards a more profound understanding of lncRNAs’ interactions in FGR-affected male and female placentas.

Recent studies revealed that lncRNAs have huge potential to regulate eukaryotic gene expression at every level [33] and control various physiological processes, including development, differentiation and other biological mechanisms [34]. There may also be some differences in placental gene expression depending on the sex of the fetus [30,31] and thus, it seems reasonable to study the lncRNA profile in various pathologies in terms of the fetus’s sex. Therefore, this study analyzed if there were sex-based differences in the lncRNA profile of the physiological and FGR-complicated human placenta. Advanced bioinformatics may reveal possible changes in the lncRNA profile that disrupt expression regulation and affect the proper development of the placenta and fetus.

## 2. Materials and Methods

### 2.1. Ethics Statement

Placental samples were collected at the Clinical Ward for Gynecology, Obstetrics, and Oncological Gynecology at the Regional Specialist Hospital in Olsztyn, according to the consent of the Bioethics Committee of the Warmia-Mazury Medical Chamber (OIL. 164/15/Bioet) in Olsztyn, Poland. All patients gave informed consent (confirmed by signature) to participate in the study.

### 2.2. Clinical Characteristics of Placental Samples

To investigate differences in the ncRNA expression profile between growth-restricted (FGR) and properly growing (control) fetuses, placentas from 6 women were collected for the FGR and control (*n* = 6) groups (Table 1). Healthy patients were added to the control group (*n* = 6) if they had a physiological pregnancy course with no clinically abnormal signs of gestation, normal fetus growth and development, and underwent a scheduled caesarean section (CS) at full-term pregnancy (37–39 weeks of gestation). Placentas for the study group (*n* = 6) were collected from patients with diagnosed asymmetric FGR (chromosomal abnormalities were excluded) who underwent scheduled (due to breech presentation, psychiatric indications or state after previous CS) or immediate CS due to the symptoms of fetal circulation insufficiency. FGR was diagnosed according to the Fetal Growth Calculator (https://medicinafetalbarcelona.org/calc/ accessed on 18 January 2019) guidelines.

The percentiles were calculated based on estimated fetal weight (EFW), biparietal diameter, head circumference, abdominal circumference and femur length, which were appointed ultrasonographically, and the values of pulsatile index flow into a medial cerebral artery, the umbilical artery and maternal uterine arteries; resistance index and systolic/diastolic ratio (GE Voluson 730). Ultrasound EFW below the 10th centile was a prerequisite to recognize abnormal fetal growth [36]. Additionally, to diagnose FGR, one of the following conditions had to be met: vessel flow estimated as poor, a cerebroplacental ratio below the 5th percentile, mean uterine artery pulsatility index higher than the 95th percentile, or correct flows but with a third percentile EFW for a given gestational age. Gestational age was assessed by the first day of the last menstrual period confirmed by ultrasound scans with crown–rump length measurement performed between the 8th and 12th weeks in those with 28-day cycles, or only by ultrasound scans between 8 and 10 weeks of pregnancy in women with irregular cycles. To investigate sex biases in the lncRNA expression profile, placental samples were derived from both fetus sexes. Placental tissue samples were collected according to established guidelines [37]. Briefly, immediately after delivery, full-thickness pieces from the middle region of the placenta, close to the umbilical cord insertion, were harvested, rinsed in sterile cold PBS buffer and dabbed dry, then snap-frozen in liquid nitrogen. Preserved tissues were stored at −70 C until RNA extraction.

### 2.3. Library Preparation and High-Throughput Sequencing

Total RNA was isolated from the placental tissues using the Qiagen RNeasy Kit according to the manufacturer’s recommendations, and DNase (Qiagen Venlo, The Netherlands) digestion was performed to obtain high-quality RNA. The quantity and integrity of the total RNA were evaluated by Quant-IT RiboGreen (Invitrogen, Waltham, MA, USA) and TapeStation RNA screen tape (Agilent Technologies, Waldbronn, Germany), respectively. For RNA library construction, only high-quality samples (RIN > 8.0) were used. Sequencing libraries were prepared with 1 μg of total RNA from each sample with the use of the Illumina TruSeq mRNA Sample Prep kit (Illumina Inc., San Diego, CA, USA). Initially, poly-T-attached magnetic beads were used to purify the poly-A-containing mRNA. Next, purified mRNA was fragmented into small pieces using divalent cations under an elevated temperature. The cleaved RNA fragments were copied into first-strand cDNA using SuperScript II reverse transcriptase (Invitrogen) and random primers, and second-strand cDNA was synthesized using DNA Polymerase I and RNase H. Further, an end repair process included the addition of a single ‘A’ base and then ligation of the indexing adapters. After purification and PCR enrichment, final cDNA libraries were created. A KAPA Library Quantification kit (Illumina Sequencing platform) for qPCR and The TapeStation D1000 ScreenTape (Agilent Technologies, Waldbronn, Germany) were applied to quantify the examined libraries. Finally, the indexed libraries were sequenced on the HiSeq4000 platform (Illumina, San Diego, USA by the Macrogen Incorporated).

### 2.4. Quality Control, Mapping and Differentially Expressed Analysis

**Figure 1:**
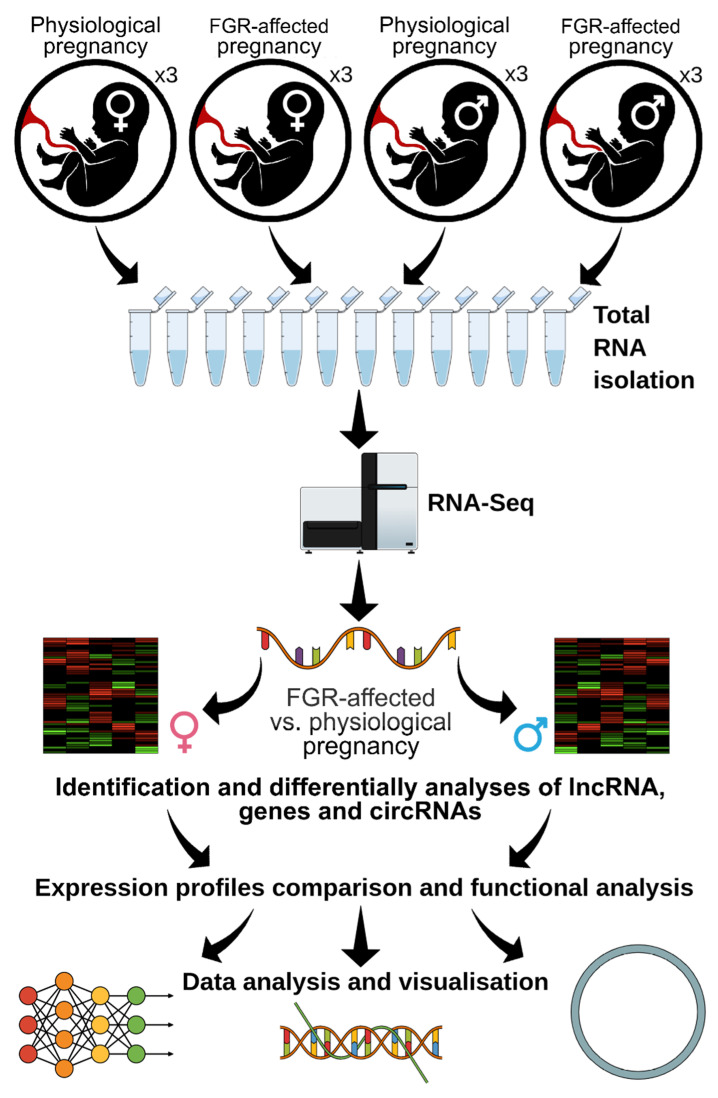
Scheme of the project.

FastQC v. 0.11.7 [38] software was used to prepare a quality control report. Trimmomatic v. 0.32 [39] was applied to eliminate adaptors and to trim reads with a low Phred score (cutoff < 20). Reads trimmed to 90 bp were splice-aware mapped to the reference human genome (GRCh38) with ENSEMBL annotation (GRCh38.90). Paired-end reads were aligned to the genome by a compilation of two methods STAR v. 2.7.1a and StringTie v. 1.3.3 [40]. Only uniquely mapped reads in the format of a BAM file were retrieved by conversion with a MarkDuplicates Picard tool (http://broadinstitute.github.io/picard/ accessed on 4 February 2020). Expression analysis was performed using the Cufflinks v. 2.2.1 pipeline [41]. The genome-wide transcriptome comparison of FGR-affected and control placentas was divided into two parts (Figure 1): FGR vs. controls in the placentas of male fetuses (mf-FGR) and FGR vs. controls in the placentas of female fetuses (ff-FGR). Using a binomial statistical test (with a cutoff of *p*_adj_ < 0.05), differentially expressed transcripts were retrieved for each comparison. Among the differentially expressed TARs, DEGs and lncRNAs were selected. Candidate DEGs were plotted in an MA and heatmap diagrams with gplots [42] and ggplot2 [43] in the R Bioconductor packages (www.r-project.org accessed on 22 February 2021). The obtained DEGs were annotated by the Gene Ontology (GO) database using g.Profiler [44] software with the *g:SCS* algorithm (*p*-value < 0.05). All in silico analyses were computed on the server (46-core CPU and 136 GB RAM) of the Regional IT Center of University of Warmia and Mazury in Olsztyn.

### 2.5. Identification of Known and Novel lncRNAs

**Figure 2:**
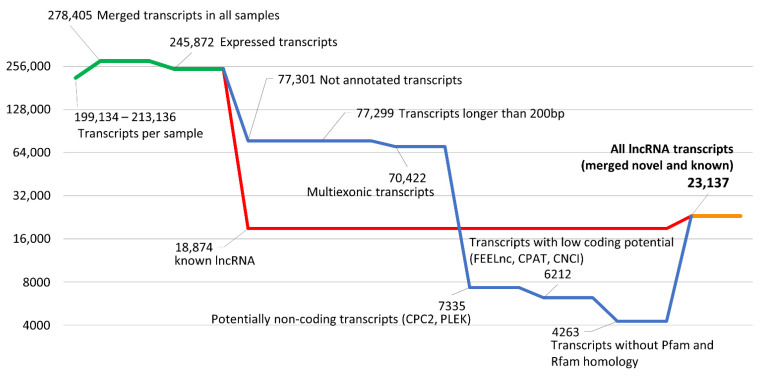
The stages of the lncRNA identification process. The Y-axis shows the number of transcripts in log_2_ scale. The green line presents all the transcripts merged from all samples. The red line describes the number of all known (annotated in GENCODE) lncRNAs expressed in analyzed samples. The blue line shows the progress of lncRNA prediction. The orange line represents the sum of detected known and novel lncRNAs.

A multistage pipeline (Figure 1 and Figure 2) was adapted for the identification of known and novel lncRNA candidates in human FGR-affected and control libraries. The transcripts of each sample were merged and annotated using references from both the ENSEMBL and GENCODE (Release 33-GRCh38.p13) databases (green line in Figure 2). Transcripts classified as the lncRNA biotype were separated as “known lncRNA” and were directly transferred to the final lncRNAs dataset (red line in Figure 2). Sequences without any annotation were classified as “not annotated” and were used in the next steps of lncRNA prediction (blue line in Figure 2). Sequences shorter than 200 bp and 1-exon transcripts were filtered out. Further, the coding potential was calculated using 7 tools in 3 different approaches. The first approach included CPC2 v. 0.1 [45] and PLEK v.1.2.4 [46], which did not require a reference genome. The second was based on FEELnc v. 0.1.1 [47] (estimated coding probability cutoff = 0.3934), CPAT v. 2.0.0 [48] and CNCI v.2 [49] (with a coding probability threshold of 0.364), tools that required a reference genome. In the third, intersected transcripts from the above approaches were scanned with the Pfam v.32.0 [50] and Rfam v.14.1 [51,52] databases. Sequences for which any homolog was found in the protein or RNA domain databases (except families of domains typical for human lncRNA) were filtered out. Finally, surviving sequences that met all the above criteria were classified as “novel lncRNA”. Both datasets of known and novel lncRNAs were combined into the final lncRNAs set used in this research (orange line in Figure 2).

### 2.6. LncRNA Target Functional Network Analyses

Among the identified lncRNAs, significant DELs (cutoff *q*-value < 0.05) were retrieved from differentially expressed TARs. *Cis*- and *trans*-acting lncRNAs were investigated according to their expression profiles and localization concerning their target protein-coding genes. Based on Cufflinks values, the Pearson correlation coefficient between lncRNAs and mRNA expression profiles was calculated. The positive and negative mutual *trans*-interactions were qualified as significant when the correlation coefficient was higher than the absolute value of 0.9 and its *p*-value was lower than 0.05. *Cis*-acting lncRNA regulatory elements were classified concerning the distance (<10 kbp) to the target gene. ToppCluster [53] was used to identify functional lncRNA–target gene interactions. The network of functional metadata for selected DELs and DEGs was generated using Cytoscape [54].

### 2.7. Prediction of Circular Organization of Transcripts

To identify circular RNA molecules in the RNA-Seq data, mapping of previously processed reads against the reference human genome (GRCh38) using the STAR v. 2.7.1a tool was performed again with the additional input parameters recommended by Jakobi et al. (S2019). The multi-modular Circtools software v. 1.1.0.8 [55] was applied for computational circRNA analyses. CircRNA detection in datasets prepared from ff-FGR, mf-FGR and both control groups was based on the DCC tool v.0.4.8 [56]. The internal circRNA structure reconstruction was conducted through the use of FUCHS [57]. Statistical tests of host gene-independent circRNA expression differences were performed in comparisons of FGR vs. controls in female and male fetuses, using the R environment-dependent CircTest package [56]. The threshold of statistical significance of differentially expressed circRNAs was set at *p* < 0.05. The required datasets containing the location of repetitive regions and individual exons in the reference genome were generated using the UCSC Genome Browser [58].

### 2.8. Validation of RNA-Seq Results Using External Transcriptomic Datasets

Data obtained regarding known and novel lncRNAs, as well as DEGs, were validated by comparison with external data generated in similar studies. The available databases were searched to select projects including placental transcriptomic data from physiological and FGR-affected pregnancies. Data from the most accurate microarray (ID: GSE147776) and RNA-Seq (ID: GSE114691) projects were chosen for further analysis. The raw data were then processed with the same approach and parameters that were applied to our data analysis. Expression values for detected DELs and DEGs were merged and compared with values obtained for datasets from the aforementioned projects.

### 2.9. Validation of RNA-Seq Results Using Quantitative Real-Time PCR (RT-qPCR)

Laboratory validation was performed using the same RNA samples as were used for RNA-Seq. Genes for validation were selected among differentially expressed lncRNAs characteristic for both comparisons (FGR vs. controls in placentas of male fetuses; FGR vs. controls in placentas of female fetuses) that were classified as known lncRNAs in the ENSEMBL database. The Enhanced Avian HS RT-PCR Kit (Sigma Aldrich, St. Louis, MO, USA) was used according to the manufacturer’s recommendations to obtain a cDNA template for quantitative real-time PCR (RT-qPCR). Expression levels of *UCA1*, *AC244205*, *HAND2-AS*, and *ACTA2-AS1* were detected by RT-qPCR using TaqMan Gene Expression Assays (*UCA1*: Hs01909129; *AC244205*: Hs01692073; *HAND2-AS*: Hs01043065; *ACTA2-AS1*: Hs04406862) (all Applied Biosystems, USA). The RT-qPCR was performed with the use of Applied Biosystems TaqMan Fast Advanced Master Mix (Thermo Fisher Scientific, Vilnius, Lithuania) according to the manufacturer’s protocol. In brief, each reaction contained 10 μL of Master Mix (2×), 1 μL of TaqMan Assay (20×), 25 ng of the cDNA template and an appropriate volume of nuclease-free water to achieve a final volume of 20 μL. The reactions were performed in four replicates in the QuantStudio 3 Real-Time PCR System (Applied Biosystems, Thermo Fisher Scientific Inc., Waltham, MA, USA). The reaction conditions were as follows: 50 °C for 2 min, 95 °C for 2 min, and 40 cycles of 1 s denaturation at 95 °C and 20 s of annealing/extension at 60 °C. Relative expression levels of target lncRNA were determined using the comparative Pfaffl method [59], where the expression was presented as the fold change relative to the control, as well as normalized to an endogenous reference gene (relative quantification RQ = 1) (*GAPDH*: Hs02786624; Applied Biosystems, Waltham, MA, USA). The results were expressed as means ± standard deviations. Statistical analysis was performed using Student’s *t*-test (two-tailed) in Prism 8 software (GraphPad Software Inc., San Diego, CA, USA). *P*-values were considered statistically significant at *p* < 0.05 (***).

## 3. Results

### 3.1. Mapping and Clustering of FGR-Affected RNA-Seq Libraries

High-throughput sequencing of the control (*n* = 6) and study group (*n* = 6) generated 660,603,254 raw paired-end reads; among these, 577,078,812 reads passed the quality control procedure of PHRED33 >20 and minimum (90 bp) sequence length filtration. The 252,001,839 paired-end reads were uniquely mapped to the reference human genome. The following mean distribution of mapped reads was obtained: 55% were derived from coding regions, 35% from untranslated regions (UTR), 2% from intergenic regions and 8% from introns. All unannotated transcripts (9.5%) were localized on chromosome 1, while 3% were on the sex chromosomes; out of these, 2307 were on the X and 85 on the Y.

### 3.2. Identification of lncRNAs in FGR-Affected Placentas

The annotated transcripts were divided into two main categories: known and novel. The first dataset comprised 80,837 protein-coding and 18,874 known long non-coding transcripts (red line in Figure 2), while the second dataset contained 77,301 expressed and novel (unannotated) transcripts. The 70,422 multi-exon transcripts with sequences longer than 200 bp were scanned for potential coding probability. The intersecting part of the five algorithms’ results encompassed 6212 transcripts without protein-coding potential. Furthermore, the Rfam and Pfam databases were searched and 1949 RNAs were excluded (blue line in Figure 1). The final dataset included 18,874 known and 4263 novel placental long non-coding RNAs (lncRNAs) transcripts that were combined in the common dataset of 23,137 lncRNAs (orange line in Figure 2).

The dataset of identified lncRNAs was characterized by an average length of 1379 bp (median 773 bp), a mean exon number of 3.2 (median 3), a mean exon length of 429.9 and the mean of expression values (FPKM) equal to 0.80819. According to the ENSEMBL, the most frequent biotypes of known long non-coding RNA annotations (85.5%) were: antisense RNA (8295 transcripts) and lincRNA (7894 transcripts). A detailed classification is provided in Table 2).

### 3.3. DEGs and lncRNAs in a Female Fetus Affected by FGR (ff-FGR)

**Figure 3:**
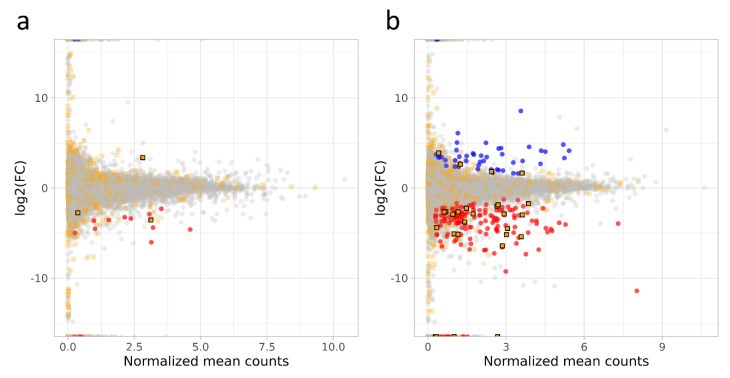
MA plot of ff-FGR (**a**) and mf-FGR (**b**) samples. Each point represents a locus (XLOC), gray dots are genes and orange rectangles represent both predicted and known lncRNAs. Blue dots are upregulated DEGs, red dots are downregulated DEGs and orange rectangles with black frames are DELs.

Differentially expressed transcripts (Figure 3a) for both genes (DEGs) and lncRNAs (DELs) were analyzed according to the normalized read counts, set significance (*q*-value < 0.05) and fold change (absolute log_2_ fold change > 1).

In total, 19 transcriptionally active regions (TARs) were differentially expressed in FGR-affected female fetus placentas, of which five were identified as DELs (two novel: *XLOC_053187*, *XLOC_056099*; three known: *ACTA2-AS1*, *PVT1* and *AL390726*). Among the remaining TARs, 12 were protein-coding genes and all of them were downregulated (Table 3, Table S1).

**Figure 4:**
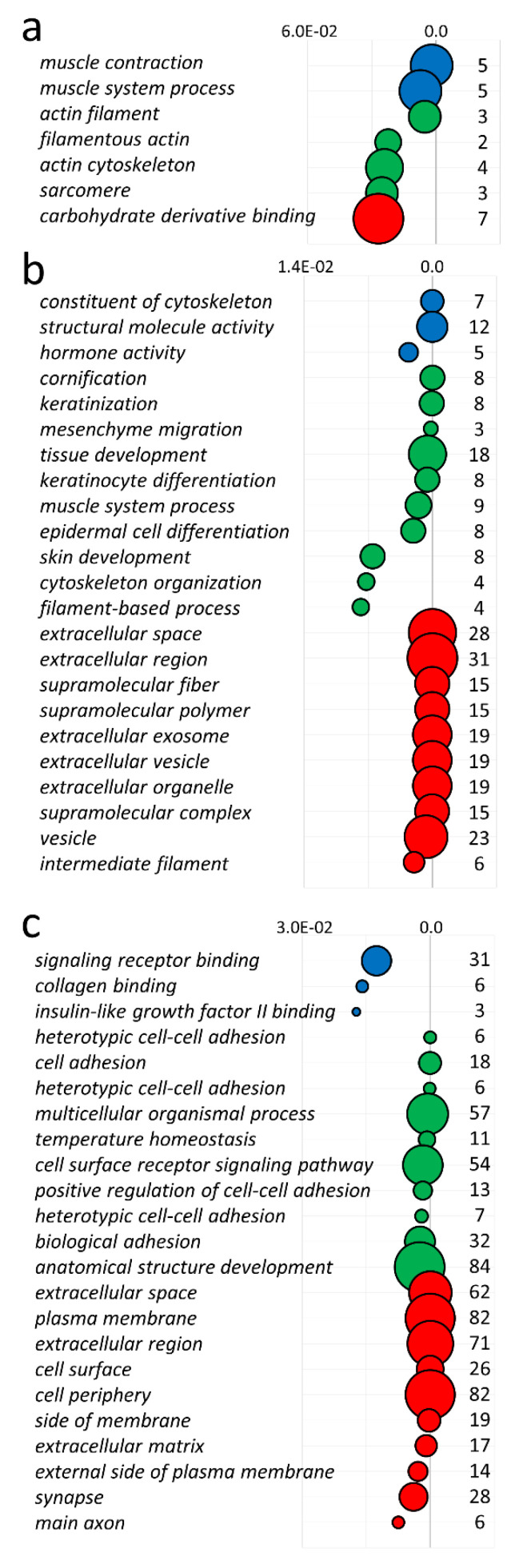
Gene Ontology enrichment plot of the top 10 categories, classified by *p*-values (x-axis of the plots), of downregulated ff-FGR (**a**), and upregulated (**b**) and downregulated (**c**) mf-FGR genes. The circles represent terms described along the y-axis, colors reflect the GO classes: blue—molecular function, green—biological process, red—cellular component. Each circle’s area corresponds to the number of genes (numerical values near to circles) enriched in each term.

Gene ontology (GO) enrichment analysis reflected the functional annotations of the seven DEGs that were qualified as two biological processes (BP), four cellular components (CC) and one molecular function (MF). In the BP category, the ‘muscle contraction’ and ‘muscle system process’ were enriched. DEGs (*MYO1A*, *ACTC1*, *PDLIM3*, *MYH3*) were then assigned to cellular components (including the ‘actin cytoskeleton’, ‘sarcomere’ and ‘actin filament’ terms). The most relevant genes (*SULF1*, *MYH3*, *TAC3*) were engaged in reproductive biology, especially playing a crucial role in the regulation of vascular endothelial growth factor production, postnatal growth retardation and female pregnancy. The top 10 terms of GO enrichment are presented in Figure 4a and all results are shown in Table 4.

### 3.4. DEGs and lncRNAs in a Male Fetus Affected by FGR (mf-FGR)

Meanwhile, 232 TARs were significantly modulated, according to their expression values, in mf-FGR (Figure 3b, Tables S2 and S3). Among all TARs, 33 encompassed novel lncRNAs and 176 known lncRNAs. In sequence, 52 DEGs were upregulated and 180 displayed decreased expression in mf-FGR. Upregulated TARs were significantly enriched in 14 BP, 3 MF and 19 CC functional association networks (Table S4 and Figure 4b). The overexpressed chorionic gonadotropin family (*CGB1*, *CGB5*, *CGB8*), *AC008687.1* and leptin (*LEP*) were significantly enriched in ‘hormone activity’. Further, ‘tissue development’ and ‘muscle contraction’ were represented by 18 (*SORBS2*, *SERPINB7*, *COL11A1*, *DSG3*, *SPRR3*, *HAND1*, etc.) and 7 (*NPNT*, *DES*, *CNN1*, *MYH14*, *ACTG2*, *ACTC1*, *ACTA1*) upregulated DEGs, respectively. Downregulated genes were classified to 40 BP, 3 MF and 14 CC relations (Table S5 and Figure 4c). The identified DEGs were expressively assigned to ‘anatomical structure development’, ‘neuron differentiation‘, ‘leukocyte migration’, ‘immune response’ and ‘primary immunodeficiency’, etc. Leukocyte migration as a part of immune reactions was displayed by downregulated genes (*IL1B*, *RET*, *CCL8*, *THY1*, *LCK*, *WNT5A*, *CYP7B1*, *CXCR4*, *CD44*). Both reproductive and immune systemic reactions were under the control of underexpressed genes (*LCN2*, *ALOX15*, *IL15*, *IL7R*, *CRISP3*). Neuronal differentiation as a complex developmental process was also significantly enriched with multiple genes (*CNR1*, *HOXD10*, *SPOCK1*, *DKK1*, *DTX1*, *MAP1A*, *CDH11*, *WNT16*, etc.).

### 3.5. lncRNA—Target Gene Relationships

The co-expression analysis of DELs vs. DEGs in ff-FGR placentas indicated one lncRNA (*ACTA2-AS1*) that was significantly correlated with five DEGs (Table 5). Trans-correlation analysis of mf-FGR placentas revealed 25 TARs associated with DELs correlated with 157 unique DEGs (Table S6). Five DELs demonstrated a high correlation coefficient (*r* > 0.9) and the most frequent ones were linked with 133 and 134 target DEGs. In total, 5 and 1623 *trans*-relations were detected in the ff-FGR and mf-FGR libraries, respectively. Based on the genomic localization, 20 lncRNAs were identified as potential *cis*-regulators of seven target genes in ff-FGR, where four relations were intergenic and 39 were intragenic (Table S7). In mf-FGR, 77 lncRNA transcripts were in a location close to 28 target genes (Table S8).

**Figure 5:**
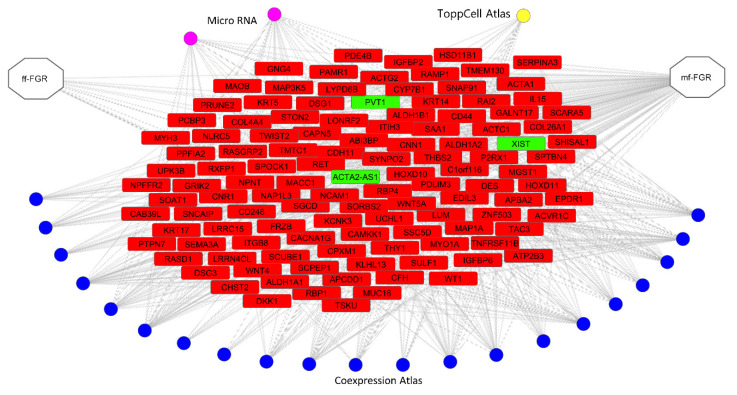
The relationship network enriched with ToppCluster. The presented relations were selected from three categories: miRNA (purple circles), the Coexpression Atlas (blue circles) and the ToppCell Atlas (yellow circles). Relations for genes (red rectangles) and lncRNAs (green rectangles) present a common part of the whole ToppCluster enrichment for both the ff-FGR and mf-FGR sets of genes.

ToppCluster was used to analyze direct and indirect relations such as co-expression and miRNA interactions, according to data available in PubMed. The relations common to both sets of mf- and ff-FGR, selected from three databases (Coexpression Atlas, ToppCell Atlas and microRNA), were visualized as a functional Cytoscape network (Figure 5; Table S9).

### 3.6. Circular Organization of Transcripts

Sequencing data analysis of ff- and mf-FGR placentas, revealed seven and three backsplicing circRNAs processes, respectively. Of the detected circRNA cases, two macromolecules were detected in both sexes: the first one in the range of the intron of the *SEPT14* pseudogene (chr1:629,675–629,725) and second within the first exon of *H19* lncRNA (chr11:1,997,424–1,997,475). All circRNAs, for which the host gene was *H19*, were encoded within its first exon, and two of them were formed by an alternative splicing process. Data obtained during the circRNAs detection workflow are presented in Table 6. A comparison of ff-FGR vs. mf-FGR did not reveal any significant differences in circRNA expression.

### 3.7. Validation of RNA-Seq Results Using External Transcriptomic Datasets

**Figure 6:**
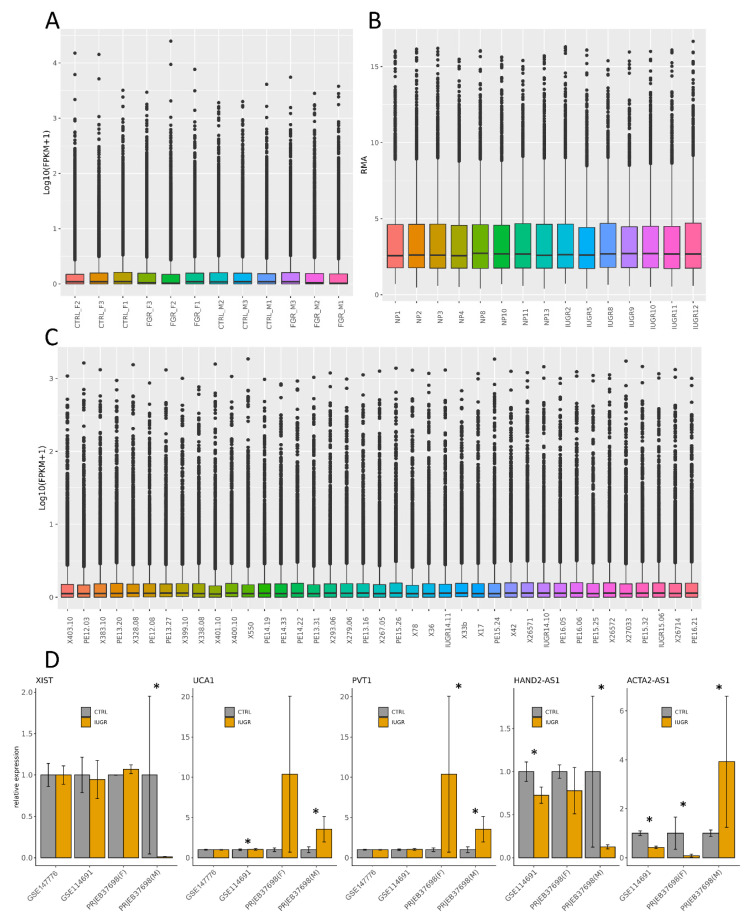
General expression profiles of samples from (**A**) the current study (PRJEB37698), (**B**) microarray (GSE14776) and (**C**) RNA-Seq (GSE114691) projects, and (**D**) relative expression profiles of the selected lncRNAs were computed based on the mean FPKM values (GSE114691 and PRJEB37698) and RMA (GSE14776). Error bars represent the SEM value. Stars (*) indicate confirmed statistically significant differentially expressed loci.

Validation with external data confirmed the presence and expression tendencies of the detected DELs and DEGs. Chosen placental transcriptomic data regarding FGR-affected and physiological samples from the microarray (Figure 6b; ID: GSE147776) and RNA-Seq (Figure 6c; ID: GSE114691) projects were normalized in log_10_(FPKM + 1) and RMA units, respectively. The general expression patterns (Figure 6a–c), as well as the expression values for specific loci, were compared (Figure 6d). Expression data showed high homogeneity, both within the projects (between samples) and between the compared projects. As the results obtained for external data were largely consistent with our results, this indicates the reliability of the performed analyses.

### 3.8. Validation of RNA-Seq Results Using Quantitative Real-Time PCR (RT-qPCR)

**Figure 7:**
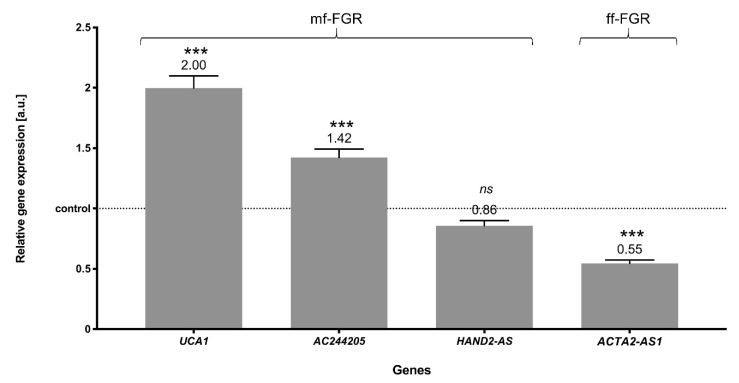
The expression levels of selected lncRNA obtained by RT-qPCR. The control is shown as normalized to a value of 1, and the samples’ expression levels indicate the changes relative to the control. The exact values of expression [a.u.] are above the bars. *p*-values were considered statistically significant at *p* < 0.05 (***).

To validate the RNA-Seq results, lncRNA with detected significantly different expression were used for RT-qPCR. Statistical analysis using the Pfaffl method [59] proved the significant changes in the expression levels of three lncRNA compared with the control (Figure 7). The results showed that two lncRNAs, *UCA1* and *AC244205*, were upregulated and one, ACTA2-AS1, was downregulated. The expression level of *HAND2-AS* was not statistically significant. The expression profiles of *UCA1*, *HAND2-AS* and *ACTA2-AS1* determined by RT-PCR were similar to those obtained in the sequencing experiment.

## 4. Discussion

Among the diversified molecules involved in the regulation of eukaryotic gene expression, lncRNAs may coordinate physiological processes, and their dysfunction may have an impact on the process of pathologies and diseases [60,61]. The mode in which lncRNAs act is multifarious and involves binding with DNA, RNA and proteins to regulate their function by affecting activation, expression level or its inhibition. [62]. To date, lncRNAs have mainly been investigated in various cancers [63] but currently, due to their confirmed regulatory potential, have been more often studied in other diseases and reported to be involved in physiological and complicated pregnancies [30,61,64,65,66]. The mechanisms leading to FGR are not well defined and attempts to identify the regulatory elements linked with this disorder have been limited to single lncRNAs [32,67]. Thus, the results obtained in this study are the first that describe the global pattern of lncRNA expression and indicate potential target genes in a case–control study of FGR.

In the current study, we evaluated the physiological and FGR-affected placentas of female and male fetuses separately. Sex-biased placental gene expression is still under investigation [19,68], so the genetic background of FGR pathology may also vary between the sexes. However, this does not exclude the possibility that some of the dysregulated pathways may lead to FGR development in either female or male fetuses. Thus, to fully investigate FGR pathophysiology, both approaches to comparing placentas, within and between the sexes, should be performed. To validate the results of the current study, the obtained datasets were compared with recent similar projects [69,70]. In general, the expression tendencies were comparable, but the aforementioned projects lacked crucial information concerning the fetal sex of each sample. A collation of analyses performed between sexes and in the cohort group additionally underlined the need for both approaches to investigating the FGR background.

In placentas from female fetuses, we identified five lncRNAs and 12 genes that were differentially expressed due to the FGR. Among the detected DELs, *ACTA2-AS1* was strongly downregulated (log_2_FC = −3.55) and indicated positive co-expression with potential target genes: *ACTC1*, *PDLIM3*, *SULF1*. According to the NONCODE database expression profile, *ACTA2-AS1* is highly expressed in the placenta compared with the other tissues. In the ff-FGR group, the co-expression Atlas database revealed the association of *ACTA2-AS1* with several DEGs (*PDLIM3*, *SULF1*, *TAC3*, *THBS2*), which may affect angiogenesis, vasculature or blood circulation, which are crucial during pregnancy.

*ACTC1* encodes Actin Alpha Cardiac Muscle 1 and is classified as highly conserved proteins involved in various types of cell motility [71]. A lack of *ACTC1* may induce apoptosis, which plays a crucial role in embryological development and may also lead to disruption of organ differentiation, specifically defects associated with heart diseases [72,73]. *PDLIM3*, involved in the determination of pregnancy-associated cardiomyopathy (PAC) [74], is potentially regulated by *ACTA2-AS1*. Besides inflammatory, immunologic and environmental factors, PAC may be triggered by a mutation in *PDLIM3* [75]. Genes known to be associated with heart diseases may also influence FGR determination, especially as fetal growth depends on the functional capacity of the placenta to transfer nutrients and oxygen from the mother to the fetus. It cannot be excluded that the downregulation of *ACTC1* and *PDLIM3* in the placenta may result in an insufficiency in substrate supply to the fetus. Nutrient supply below the demand prevents the fetus from achieving its genetic growth potential and leads to FGR [76]. A high lncRNA–gene co-expression coefficient (0.93) between *ACTA2-AS1* and downregulated *SULF1* (log_2_FC = −4.39) in ff-FGR was also detected. The function of the protein coded by *SULF1* is to release 6-O-sulfate groups from the heparan sulfate, which, in consequence, modifies the growth factor-binding sites in proteoglycans [77]. Therefore, sulfatase plays a major role in many important processes, such as angiogenesis, cell signaling and embryogenesis [78,79]. Research performed in mice revealed that *SULF* mutations are responsible for brain and skeletal malformations, abnormal innervations of smooth muscle and even embryonic lethality [80,81]. Multiple *SULF1* effects arise from the modulation of the BMP, Hedgehog and Wnt signaling pathways, as well as fibroblast growth factors. An association between *SULF1* mutation and occurrences of recurrent miscarriage has also been postulated [82]. Thus, the detected changes in *SULF1* expression patterns between physiological and FGR-complicated placentas may play an important role in the determination of this pregnancy pathology.

Single-cell RNA-Seq showed that tachykinin 3 (*TAC3*) was upregulated in first- and second-trimester placentas, specifically in cells of extravillous trophoblasts and cytotrophoblasts [83]. Additionally, dysregulation of *TAC3* may be implicated in PE, pregnancy-related hypertension and, therefore, in adverse pregnancy outcomes [84]. Therefore, underexpression of *TAC3* (log_2_FC = −5.04) in the third-trimester placenta may be specific to FGR occurrence. In the placentas of the patients with PE, significantly increased expression levels of *THBS2* are mediated by miRNAs and influence trophoblast growth, invasion, migration and cell apoptosis suppression [85]. Our research revealed positive expression associations between *ACTA2-AS1* and both *TAC3* (log_2_FC= −4.6) and *THBS2* (log_2_FC = −3.24) in ff-FGR. Enrichment analysis revealed a link between *ACTA2-AS1* and upregulated *PVT1* (log_2_FC = 3.4), also known as a lncRNA. The reverse tendency, downregulation of *PVT1*, was observed in gestational diabetes mellitus (GDM) and PE placentas [86]. *PVT1* knockdown significantly promotes apoptosis, and inhibits the proliferation, migration and invasion of trophoblast cells; its overexpression contributes the opposite effects. *PVT1* affects numerous miRNAs and genes engaged in maintaining the physiological action of the placenta [66,86]. Thus, *PVT1*, considered to be an important oncogene, may also be a critical lncRNA regulator in placenta physiology and therefore pregnancy diseases. A metadata analysis showed associations between *PVT1* and *MYH3*. Research performed in the rat model for PE revealed that *MYH3*, *MYH8* and *TNNI1* were enriched in the ‘striated muscle contraction’ pathway [87]. The involvement of myosins, major contractile proteins, in the pathophysiology of experimental PE [87] may suggest that dysregulation of *MYH3* expression is also associated with other pregnancy disorders. One of the conditions required to diagnose FGR is an estimation of vessel flow as poor or a cerebroplacental ratio below the fifth percentile or a mean uterine artery pulsatility index higher than the 95th percentile [31]. In our studies, the *MYH3* expression pattern was determined as significantly downregulated (log_2_FC= −3.5), which may be directly associated with reduced placental vessel flow underlying the FGR pathophysiology. Our previous investigation of FGR placentas [31] revealed that some DEGs crucial for pregnancy development may be involved in the pathophysiology of different pregnancy disorders in PE samples. The identified ff-FGR DEGs may be correlated with the expression patterns of regulatory elements such as *ACTA2-AS1*, which significantly emphasize the validity of lncRNAs in the course of pregnancy.

In male fetuses’ placentas, among the 43 detected DELs, 17 transcripts were classified as a novel. Based on their close distance, 28 potential target genes were identified, and a co-expression analysis revealed 1511 significant relationships with DELs. One mf-FGR DEL, *AC092017.4*, displayed enhanced regulative potential of 134 *trans*-target genes. Positive correlations were measured between *AC092017.4* (log_2_FC= −5.09) and solute carrier family genes (*SLC1A2* and *SLC38A5*), which were also downregulated (log_2_FC= −3.80 and log_2_FC= −2.72, respectively). However, in mf-FGR placentas, another SLC family member, *SLC22A2*, was upregulated (log_2_FC= 3.40). Among the diverse SLC family, which specializes in transmembrane transport, the members *SLC1A2*, *SLC38A5* and *SLC22A2* encode glutamate [88], sodium-coupled neutral amino acid [89] and polyspecific organic cation [90] transporters, respectively. OCT2, a product of *SLC22A2*, is responsible for the re-uptake of norepinephrine and serotonin from the extracellular fluid, which regulates vasoconstriction and blood flow across the placenta to the fetus [91]. Previous studies indicated that underexpression of the amino acid transport system precedes FGR [64,66]. Generally, it is known that altered placental transport of essential nutrients and molecules directly contributes to FGR [92,93,94] but the contributing regulatory mechanisms remain unknown. The obtained results may suggest that dysregulation of transporters is an effect of *AC092017.4*’s action in mf-FGR placentas, and its outcome concerns insufficient nutrient supply to the placenta and fetus. Moreover, *AC092017.4* is positively correlated with DEGs (*IL1B*, *RET*, *CCL8*, *WNT5A*, *CYP7B1*, *ALOX15*, and *IL15*) assigned to immune reactions. Animal models demonstrated that obesity and excessive nutrition increased the concentrations of IL1B and other proinflammatory cytokines, which resulted in the development of insulin resistance and inordinate fetal growth [95,96]. Conversely, we can presume that fetal malnutrition and growth deficiency in the course of FGR may be a consequence of decreasing *IL1B* (log2FC = −5.11) expression. *Il15* encodes a cytokine required for NK-cell lineage development. Studies of a Il15-deficient rat model revealed a lack of uterine NK (uNK) cells, which normally regulate blood flow to the placenta through the development of the uterine vasculature [97]. In humans, changes in uNK cell number or activity lead to failure of the uterine vascular system [98,99] and notably disturb fetal growth [100]. The uNK cells synthesize immunoregulatory cytokines, particularly IFN-γ, which significantly upregulates chemokines such as CXCL9, CXCL10, CCL8 and CCL5 [101]. A decreased level of *CCL8* expression (log_2_FC= −4.06) in mf-FGR samples may be the result of uNK and immune response dysregulation caused by DELs and DEGs, such as *AC092017.4* and *Il15*.

Another gene that may be dysregulated by *AC092017.4* is the RET proto-oncogene (*RET*) which plays a signaling role in the immune system by its receptor localized on NK, monocytes and B and T lymphocyte cells [102]. *RET* has been demonstrated to regulate pathways that are involved in cellular survival, proliferation, differentiation, migration and chemotaxis [103], which are significant processes during pregnancy. Pregnancy outcome also depends on WNT5A, and any disturbances in its signaling pathway during early pregnancy reflect defective decidualization and placentation, which, in late pregnancy, are manifested as various abnormalities [104]. *AC092017.4* may also affect the expression of enzyme genes, such as *ALOX15* and *CYP7B1*. *ALOX15* encodes a lipoxygenase responsible for resolvin and protectin formation, which function as inflammation mediators. Interestingly, in rats, *ALOX15* expression increases toward term and is higher in female fetuses’ placentas [105]. The significant underexpression of *ALOX15* (log_2_FC= −3.30) in mf-FGR placentas, accompanied by dysregulation of *CYP7B1* (log_2_FC= −2.71) and other genes involved in inflammation, additionally underlines the role of immune response pathways in this pregnancy disorder. *CYP7B1* encodes oxysterol 7α-hydroxylase, which is essential for cholesterol transformation into structurally distinct metabolites. Oxysterols have pleiotropic roles and act through diverse receptors, triggering metabolic signals to coordinate immune activity and inflammation [106,107]. *AC092017.4* may regulate *trans*-target genes responsible for the modulation of immune reactions at various levels and different modes. The mechanisms underlying spontaneous abortion, pre-term labor and pre-term pre-labor rupture of the membranes are associated with an altered inflammatory response [108]. It has been suggested that they may also be involved in FGR [31] and the currently obtained results seem to confirm that supposition. Therefore, we propose the detected lncRNAs as regulatory elements that are useful for identifying biomarkers and developing therapies to target specific molecular pathways in FGR treatment.

Among the DELs identified in mf-FGR samples, *UCA1* was upregulated (log_2_FC = 1.66) and co-expressed with *GABRP* (log_2_FC = 3.07) and *NLRP2* (log_2_FC = 1.62). *UCA1* is increased in various cancers and promotes processes such as proliferation, migration and immune escape, and inhibits apoptosis [32,109]. Although the specific role of the *UCA1* in the course of pregnancy is unknown, besides the above functions, it may be associated with *trans*-target genes such as *GABRP* and *NLRP2*, impacting placenta wellbeing. Pi, a subunit of gamma-aminobutyric acid type A receptor (*GABRP*), affects pathways during decidualization of the stromal cell [110], and also has implications in preeclampsia [111]. NLR Family Pyrin Domain Containing 2 (*NLRP2*), by impacting the caspase-1 and NF-kB pathway, is believed to modulate the immune response [112]. The female mouse model revealed that a lack of *NLRP2* manifested as more frequent early embryonic loss and developmental disruptions [113]. Thus, *NLRP2* and *GABRP*, the potential *trans*-target genes for differentially expressed *UCA1*, may be the key genes whose dysregulation leads to adverse pregnancy.

The performed global lncRNAs, which are characteristic in FGR case–control studies, were upgraded, with an analysis of the circular RNAs (circRNAs). Covalent bond linking of the 3′ and 5′ ends generated by backsplicing characterizes this novel class of non-coding RNA—circRNA [114]. It appears that circRNAs, previously considered as splicing by-products, have the potential to regulate gene expression [115], and emerging evidence indicates that circRNAs might play an important role in severe PE [116]. CircRNAs detected in the currently studied FGR placentas targeted *H19* loci, known as lncRNA. Loss of *H19* imprinting is suspected to be involved in the pathomechanism of preeclampsia and growth restriction during pregnancy [61,117]. Moreover, *H19* downregulation inhibits the TGF-β signaling pathway, which affects trophoblast cell migration and invasion, directly leading to FGR [118]. *H19* did not reveal differences in its expression profile, although our research may shed new light on novel circRNA regulations within the first exon of this lncRNA specific to the placenta. Furthermore, an analysis of mf-FGR placentas identified the circRNA encoded in the first intron of the *BACE2* gene. This may suggest that the sex-specific process of *BACE2* pre-mRNA maturation is regulated by the circRNA. During PE, placental *BACE2* expression is upregulated [119,120], which may also indicate the impact of this gene on FGR pathogenesis. The influence of circRNAs on the pre-mRNA maturation process and, therefore, on the protein content in cells, may affect abnormalities in fetal development in the long term.

In view of the previous studies, our results indicate that disturbed expression of regulatory elements and their target genes involved in various processes may be one of the major FGR causes [30]. Our current studies have revealed that the genetic background for FGR may differ between sexes. In female placentas, DELs and DEGs were associated mainly with the vasculature, while in male placentas, disturbed expression predominantly affected immune processes. Any abnormalities during angiogenesis and, further, in placental vessels flow lead to disorders in the efficient transfer of nutrients, which is essential for proper fetus growth [121]. However, disturbances in the course of the immune response are linked with several pregnancy pathologies [108]. Despite different DELs and DEGs between sexes, the outcome may be the same—a growth-restricted male or female fetus.

## 5. Conclusions

The in utero environment has profound implications for fetal development and long-term effects in childhood and later life. The mechanisms responsible for such connections are poorly understood but are likely to be modulated by gene expression and transcriptional regulatory mechanisms [122]. While FGR-complicated offspring are prone to various diseases, it cannot be excluded that this is an effect of specific DEG and DEL disruption, linked with FGR etiology. To summarize, global differences in expression between male and female lncRNAs and their potential target genes were analyzed and the possible associations underlying FGR were indicated. Nevertheless, FGR is the final phenomenon, which is caused by maternal, fetal or placental conditions, and also their combinations, and it has not been determined how much placental gene expression may affect FGR pathoetiology. Moreover, the cause–effect relationship between gene expression and FGR occurrence is still unclear. Therefore, further studies are necessary to reveal FGR’s pathophysiology.

## Figures and Tables

**Table 1:** Characteristics of the placental samples.

Biosample Number	Fetus Sex	Fetus Weight [g]	Week of Gestation	Apgar Score	Maternal Age	FGR Stage *
ERX4055529	Male	750	26	2	31	4
ERX4055530	Male	1680	30	2	37	3
ERX4055531	Male	2060	39	10	28	1
ERX4055532	Female	2360	36	10	28	1
ERX4055533	Female	2260	37	10	24	1
ERX4055534	Female	2000	37	10	27	1
ERX4055535	Male	3230	37	10	35	-
ERX4055536	Male	3420	39	10	37	-
ERX4055537	Male	3500	39	10	31	-
ERX4055538	Female	2960	39	10	23	-
ERX4055539	Female	3300	38	10	29	-
ERX4055540	Female	3100	38	10	30	-

* FGR stage was determined according to Figueras and Gratacós (2014) [35] and the Fetal Growth Calculator (https://medicinafetalbarcelona.org/calc/ accessed on 18 January 2019) guidelines. Stage 1: severe smallness or mild placental insufficiency; Stage 2: severe placental insufficiency; Stage 3: low-suspicion fetal acidosis; Stage 4: high-suspicion fetal acidosis.

**Table 2:** Overall statistics of known lncRNAs. Transcripts’ biotypes were classified according to the ENSEMBL database.

Transcript Biotypes	Transcripts
antisense_RNA	8295
lincRNA	7849
processed_transcript	484
retained_intron	441
sense_intronic	657
sense_overlapping	250
TEC	722
others	176

**Table 3:** Differentially expressed genes identified in ff-FGR samples. Normalized fold changes (log_2_ fold change) were calculated on the locus level (XLOC). Loci containing lncRNAs are marked in the “lncRNA” column as known (referring to annotated lncRNA) and novel (referring to lncRNAs identified in this study). “-” in the “lncRNA” column means that in this specific locus, lncRNA has not been detected.

Gene ID	Gene Name	Log_2_ Fold Change	ENSEMBL ID	lncRNA
XLOC_000619	-	Inf	NA	-
XLOC_006184	*ACTA2-AS1*	−3.55	ENSG00000180139	known
XLOC_006962	-	-Inf	NA	-
XLOC_013293	*MYO1A*,*TAC3*	−4.60	ENSG00000166866, ENSG00000166863	-
XLOC_013509	*PPFIA2*	−4.98	ENSG00000139220	-
XLOC_019012	*ACTC1*	−6.02	ENSG00000159251	-
XLOC_019742	-	-Inf	NA	-
XLOC_023789	*CAMKK1*,*P2RX1*	−2.88	ENSG00000004660, ENSG00000108405	-
XLOC_023922	*MYH3*	−3.55	ENSG00000109063	-
XLOC_040198	-	Inf	NA	-
XLOC_042821	*PDLIM3*	−2.30	ENSG00000154553	-
XLOC_044217	*SGCD*	−3.63	ENSG00000170624	-
XLOC_048901	*THBS2*	−3.24	ENSG00000186340	-
XLOC_051439	*TMEM130*	−4.53	ENSG00000166448	-
XLOC_052796	*SULF1*	−4.39	ENSG00000137573	-
XLOC_053187	*MIR1204*,*PVT1*, *PVT1_1*,*PVT1_3*	3.38	ENSG00000283710, ENSG00000249859, ENSG00000276443, ENSG00000278324	-, novel, known
XLOC_054578	-	-Inf	NA	-
XLOC_054962	*ALDH1B1*	−3.37	ENSG00000137124	-
XLOC_056099	*ANKRD18A*,*FAM95C*	−2.75	ENSG00000180071, ENSG00000225345, ENSG00000250989, ENSG00000273036, ENSG00000272934, ENSG00000272904, ENSG00000283486	-, novel, known

**Table 4:** The results of Gene Ontology analysis for differentially expressed genes detected in ff-FGR samples.

Term Name	Term ID	Source	*p*-Value *	Gene Names
muscle contraction	GO:0006936	BP	3.3074 × 10^−3^	*P2RX1,SULF1,ACTC1,SGCD,MYH3*
muscle system process	GO:0003012	BP	1.2096 × 10^−2^	*P2RX1,SULF1,ACTC1,SGCD,MYH3*
actin filament	GO:0005884	CC	8.5988 × 10^−3^	*PDLIM3,ACTC1,MYO1A*
filamentous actin	GO:0031941	CC	3.7185 × 10^−2^	*PDLIM3,MYO1A*
actin cytoskeleton	GO:0015629	CC	4.0095 × 10^−2^	*PDLIM3,ACTC1,MYO1A,MYH3*
sarcomere	GO:0030017	CC	4.2036 × 10^−2^	*PDLIM3,ACTC1,MYH3*
carbohydrate derivative binding	GO:0097367	MF	4.4875 × 10^−2^	*CAMKK1,P2RX1,SULF1,ACTC1,MYO1A,THBS2,MYH3*

* calculated with the g:SCS algorithm.

**Table 5:** *Trans*-relations between lncRNAs and differentially expressed genes in ff-FGR samples.

lncRNA	DEG	lncRNA Name	DEG Name	Correlation	*p*-Value
XLOC_006184	XLOC_019012	*ACTA2-AS1*	*ACTC1*	0.960	2.3481 × 10^−3^
XLOC_006184	XLOC_042821	*ACTA2-AS1*	*PDLIM3*	0.948	4.0051 × 10^−3^
XLOC_006184	XLOC_044217	*ACTA2-AS1*	*SGCD*	0.943	4.8411 × 10^−3^
XLOC_006184	XLOC_052796	*ACTA2-AS1*	*SULF1*	0.933	6.6462 × 10^−3^
XLOC_006184	XLOC_054962	*ACTA2-AS1*	*ALDH1B1*	0.976	8.6087 × 10^−4^

**Table 6:** All detected circular RNA molecules in ff-FGR and mf-FGR samples.

Sample Set	Chr	Start	End	Host Gene	Strand	Region
ff-FGR	1	629,675	629,725	*AL669831.3*	-	intron
ff-FGR	1	207,336 713	207,336,763	*CD55*	+	exon 7
ff-FGR	11	1,997,400	1,997,475	*H19*	-	exon 1
ff-FGR	11	1,997,424	1,997,475	*H19*	-	exon 1
ff-FGR	11	1,997,697	1,997,767	*H19*	-	exon 1
ff-FGR	KI270721.1	52,582	52,657	not annotated	-	intergenic
ff-FGR	KI270721.1	52,606	52,657	not annotated	+	intergenic
mf-FGR	1	629,675	629,725	*AL669831.3*	-	intron
mf-FGR	11	1,997,424	1,997,475	*H19*	-	exon 1
mf-FGR	21	41,178,852	41,178,960	*BACE2*	+	intron

## Data Availability

The sequencing data, novel lncRNA and circRNA sequences from this study are available under accession no. PRJEB37698 in the European Nucleotide Archive (https://www.ebi.ac.uk/ena/browser/view/PRJEB37698, accessed on 1 January 2021).

## Supplementary Materials

The following are available online at https://www.mdpi.com/article/10.3390/cells10040921/s1, Table S1. Differentially expressed genes identified in ff-FGR samples described separately for each transcript (the “transcripts_ff-FGR” sheet). Transcripts annotated in GENCODE as the lncRNA are marked in the “lncRNA” column as known (referring to annotated lncRNA) and novel (referring to lncRNAs identified in this study). The sheet named “isoforms_FPKM” contains the FPKM values for all transcripts (in rows) in each sample separately (in columns). Table S2. Differentially expressed genes identified in mf-FGR samples. Normalized fold changes (log_2_ fold change) were calculated on the locus level (XLOC). Loci containing lncRNAs are marked in the “lncRNA” column as known (referring to annotated lncRNA) and novel (referring to lncRNAs identified in this study). “-” in the “lncRNA” column means that in this specific locus, lncRNA has not been detected. Table S3. Differentially expressed genes in mf-FGR samples described separately for each transcript (the “transcripts_mf-FGR” sheet). Transcripts annotated in GENCODE as the lncRNA are marked in “lncRNA” column as known (referring to annotated lncRNA) and novel (referring to lncRNAs identified in this study). The sheet named “isoforms_FPKM” contains the FPKM values for all transcripts (in rows) in each sample separately (in columns). Table S4. The results of Gene Ontology analysis for upregulated genes detected in mf-FGR samples. Table S5. The results of Gene Ontology analysis for downregulated genes detected in mf-FGR samples. Table S6. *Trans-*relations between lncRNA and differentially expressed genes in mf-FGR samples. Table S7. *Cis*-relations between lncRNA and differentially expressed genes in ff-FGR samples. Table S8. *Cis*-relations between lncRNA and differentially expressed genes in mf-FGR samples. Table S9. The table of selected records from the ToppCluster enrichment analysis visualized in Figure 5. Each row is the record describing the relation/interaction between genes listed in the columns “ff_GeneSet” and “mf_GeneSet”. The “Category” column refers to the colored points in Figure 5.
